# The Effect of Empagliflozin on Renal Outcomes in Patients With Established Cardiovascular Disease: Systematic Review and Meta‐Analysis of Randomised Placebo‐Controlled Trials

**DOI:** 10.1002/edm2.70203

**Published:** 2026-04-17

**Authors:** Mansour Bahardoust, Fatemeh Naseri Rad, Sepideh Mousavi, Mohammadsadra Shamohammadi, Meisam Haghmoradi, Azin Ghaffari

**Affiliations:** ^1^ Department of Epidemiology, School of Public Health & Safety Shahid Beheshti University of Medical Sciences Tehran Iran; ^2^ School of Medicine Iran University of Medical Sciences Tehran Iran; ^3^ Pharmacy Faculty Tabriz University of Medical Sciences Tabriz Iran; ^4^ Gastrointestinal and Liver Diseases Research Center Iran University of Medical Sciences Tehran Iran; ^5^ Urmia University of Medical Sciences Urmia Iran; ^6^ Rajaie Cardiovascular Medical and Research Center Iran University of Medical Sciences Tehran Iran

**Keywords:** cardiovascular disease, chronic kidney disease, empagliflozin, renal outcomes

## Abstract

**Background:**

Empagliflozin, a sodium‐glucose cotransporter‐2 inhibitor (SGLT2i), may be associated with improved renal outcomes. However, the magnitude and potential heterogeneity of effect among patients with cardiovascular disease (CVD) remain unclear. This systematic review aimed to evaluate the efficacy and safety of empagliflozin on renal outcomes in patients with established CVD.

**Methods:**

Based on the PRISMA guideline, we searched PubMed, Embase, Scopus Google Scholar, Cochrane Library and Web of Science databases, as well as study references, to find randomised controlled clinical trials (RCTs) that evaluated the effect of empagliflozin (regardless of dose) compared with placebo on renal outcomes in patients with CVD, with no time limit, up to 20 August 2025. Outcomes included kidney disease progression, composite renal outcome, nephropathy, doubling of serum creatinine and safety.

**Results:**

Thirteen RCTs involving 36,169 patients were included. A pooled analysis of 12 studies showed that empagliflozin was significantly associated with a reduced risk of progression of kidney disease (HR: 0.66, 95% CI: 0.58, 0.74, *I* ^2^: 11.1%). Empagliflozin was also associated with a reduced risk of the composite renal outcome (HR: 0.7% 95% CI: 0.55, 0.86, *I* ^2^: 0%), diabetic nephropathy (HR: 0.59% 95% CI: 0.42, 0.75, *I* ^2^:0%) and doubling of serum creatinine in CVD patients (HR: 0.60% 95% CI: 0.41, 0.78, *I* ^2^:0%).

**Conclusion:**

Empagliflozin may significantly reduce the risk of CKD outcomes in patients with CVD compared with placebo. Empagliflozin also had a favourable safety profile. Empagliflozin can be prescribed as an effective and safe antidiabetic drug in patients with CVD to improve renal outcomes and prevent CKD progression.

## Graphical abstract

Empagliflozin may be significantly reducing the risk of CKD outcome in patients with CVD compared with placebo. Empagliflozin also had a favourable safety profile.

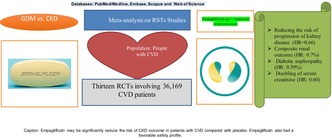

## 1 Introduction

Chronic kidney disease (CKD) and cardiovascular diseases (CVD), including heart failure (HF), are among the most common global health challenges, with their incidence and prevalence increasing in parallel with the increasing incidence of type 2 diabetes mellitus (T2DM), a significant and common cause of both [1, 2, 3]. CKD affects approximately 10% of the world's population [4]. Beyond progression to end‐stage renal disease (ESRD) requiring dialysis or transplantation, CKD is strongly associated with increased cardiovascular morbidity and mortality [5]. Approximately 40 out of 100 patients with type 2 diabetes (T2DM) have CKD, based on estimated glomerular filtration rate (eGFR) or albuminuria criteria [6, 7, 8]. Of these, more than 20% are estimated to have clinically overt CKD (eGFR < 60 mL/min/1.73 m^2^) [9, 10]. Studies have shown that a decrease in eGFR to < 60 mL/min/1.73 m^2^ is an independent risk factor for CVD, including diabetes [11, 12, 13]. Therefore, the progression of kidney disease and the risk of renal outcomes in heart disease patients may differ from those in the general population [14, 15].

For decades, inhibition of the renin–angiotensin–aldosterone system (RAAS) has remained the cornerstone therapy to retard CKD progression and decrease CV risk [16, 17]. However, a substantial residual risk of end‐stage kidney disease progression remains despite RAAS inhibition, highlighting the limitations of existing therapies in fully mitigating the cardio‐renal complications of CKD [18, 19]. Differences in the reported residual cardio‐renal risk with RAAS inhibition across studies underscore the need for new treatments to reduce further kidney and cardiovascular complications [20, 21, 22].

To address the residual risks that persist despite RAAS inhibition, sodium–glucose cotransporter‐2 (SGLT2) inhibitors have emerged as a novel therapeutic strategy with transformative cardio‐renal benefits [23]. Although developed as glucose‐lowering agents, SGLT2 inhibitors such as empagliflozin slow chronic eGFR decline, reduce albuminuria and lower rates of kidney failure, cardiovascular death and heart‐failure hospitalisation, benefits that extend to CKD populations [24, 25, 26, 27]. The EMPA‐KIDNEY trial demonstrated that empagliflozin reduced kidney disease progression, renal outcome or cardiovascular death in CVD, with benefits consistent regardless of diabetes status [28, 29, 30, 31]. Given the broad and profound cardio‐renal benefits observed, the U.S. Food and Drug Administration has approved SGLT2 inhibitors for the treatment of CKD to improve both renal and cardiovascular outcomes [32].

Empagliflozin is the most commonly prescribed SGL2 for the management of diabetic patients, with its documented cardiovascular benefit distinguishing it from other antidiabetic agents [24, 33, 34]. Empagliflozin directly affects renal tubular function and can have beneficial effects on blood pressure, body weight and heart and kidney failure, so in theory, they may be associated with improved renal outcomes in CVD patients [35]. Although its macrovascular effects have been proven, its microvascular effects remain controversial. On the other hand, its effects on the microvascular system have been investigated in several RCTs, with conflicting results [17, 36, 37, 38, 39, 40, 41]. The magnitude of the effect of empagliflozin on renal outcomes among patients with CVD has been reported to be heterogeneous in different studies, possibly due to the small sample size of the studies examined [17, 36, 37, 38, 39, 40, 41].

Although individual trials have demonstrated the efficacy of empagliflozin in slowing the progression of CKD and improving renal outcomes among people with CVD, a systematic integration of these findings will quantify the benefits and any potential risks in different populations with CKD. Therefore, we designed a systematic review and meta‐analysis to provide a comprehensive assessment of the impact of empagliflozin on renal outcomes among participants with CVD.

## 2 Methods

### 2.1 Protocol Registration and Search for Potential Studies

We conducted a systematic review and meta‐analysis in accordance with standardised reporting and methodological guidance (PRISMA (Preferred Reporting Items for Systematic Reviews and Meta‐Analyses) guidelines) [42]. A protocol for the review protocol was developed before study initiation and registered with the International Prospective Register of Systematic Reviews.

### 2.2 Literature Search

A literature search was conducted to identify randomised controlled trials (RCTs) that investigated the efficacy and safety of empagliflozin on renal outcomes in adults with CVD, using mesh terms. A search strategy was applied across multiple databases, including MEDLINE/PubMed, EMBASE, Scopus, Web of Science and the Cochrane Library, with no time limit until 22 August 2025. We also searched ClinicalTrials.gov and the World Health Organization (WHO) International Clinical Trials Registry Platform for ongoing or unpublished trials. In addition to database searches, we manually screened reference lists of relevant studies. Two independent investigators searched.

After the overall search strategy was defined based on the PICO (Patient, Intervention, Comparison and Outcome) framework, the databases were searched using terms such as ‘empagliflozin’, ‘SGLT2 inhibitors’, ‘kidney disease’, ‘renal outcome’, ‘chronic kidney disease’, ‘chronic kidney failure’, ‘Kidney Failure’,’Chronic Renal Disease’, ‘Cardiovascular Diseases’, ‘Major Adverse Cardiac Events’, ‘Cardiac Event’, ‘Cardiac Failure’, ‘Heart Failure’, ‘Heart Decompensation’, ‘Atrial fibrillation’ and ‘Myocardial Failure’.

PICO was defined as follows:

Population: People with CVD Coronary heart disease (atherosclerosis), atrial fibrillation, stroke, congenital heart disease, arrhythmias, peripheral artery disease and heart failure (HF) in RCTs, Intervention/Comparison: Receiving empagliflozin (regardless of dose) versus placebo, Outcomes: kidney disease progression, composite renal outcome, nephropathy, doubling of serum creatinine and safety.

In all included studies, the composite renal outcome was defined as time to first instance of chronic dialysis, kidney transplantation, sustained decline in eGFR ≥ 40%, or sustained eGFR less than 15 mL/min/1.73 m^2^ if baseline eGFR was above 30 mL/min/1.73 m^2^, or less than 10 mL/min/1.73 m^2^ for patients whose baseline eGFR was 30 mL/min/1.73 m^2^ or less [39]. The safety and adverse event profiles were evaluated according to a standard definition that has been validated in prior studies [43]. Documented adverse events included hypoglycemia, instances where plasma glucose levels dropped to ≤ 70 mg/dL and/or situations requiring assistance. Events related to urinary tract infections, genital tract infections, acute kidney injury, volume depletion, bone fractures and hyperkalemia were identified through a search of reported adverse events in the studies. CKD progression in the two groups was determined by the estimated glomerular filtration rate (eGFR) in the studies. CKD was defined as an eGFR < 60 mL/min/1.73 m^2^ or evidence of kidney damage for at least three months. Kidney disease progression was defined as a sustained decline in eGFR of at least 40% from randomization, development of end‐stage renal disease, sustained eGFR < 10 mL/min/1.73 m^2^, or death from renal failure [29]. Diabetic nephropathy was defined by the standard definition as persistent albuminuria (> 300 mg/day or > 200 μg/min) confirmed on at least 2 occasions 3–6 months apart, or progressive decline in glomerular filtration rate (GFR) or high arterial blood pressure [44].

### 2.3 Eligibility Criteria

Our inclusion criteria included RCTs that evaluated the effect of empagliflozin (regardless of dose) versus placebo in adult patients (≥ 18 years) with CVD (regardless of type), regardless of diabetes status, availability of an effect size for the association of empagliflozin on renal outcomes (as defined), RCTs with at least 100 patients in each treatment subgroup, RCTs with at least 1 year of follow‐up and published studies in English. RCTs that focused on end‐stage renal disease, including dialysis or transplant recipients, were excluded. Studies were also excluded if CVD patients could not be separated from other populations, unless CVD‐specific subgroup data were available. Also, review studies, studies that evaluated the effect of empagliflozin versus other SGLT2 inhibitors or other antidiabetic drugs, letters to the editor, animal/in vitro studies, case reports and observational studies were excluded from this systematic review and meta‐analysis.

### 2.4 Screening

**FIGURE 1:**
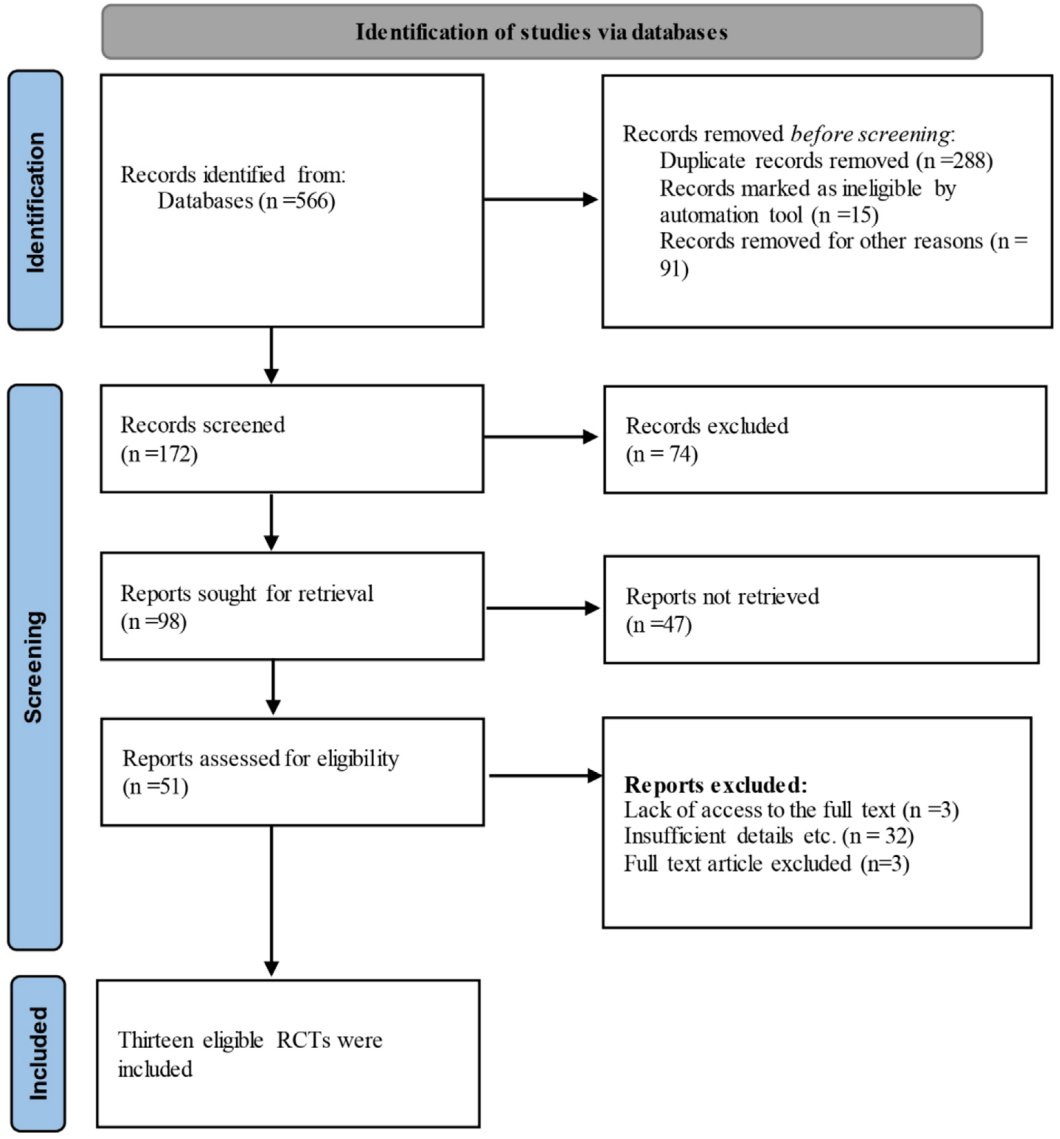
Flowchart page of studies based on PRISMA 2020.

The screening process followed a two‐stage approach. First, two reviewers independently screened titles and abstracts for eligibility based on predefined criteria. In the initial search, 586 articles were found from all databases. Using EndNote version 21 software, duplicate articles were identified and removed between sources. Irrelevant records were also removed, and potentially eligible citations were continued for full‐text review. Second, two reviewers independently assessed full‐text articles against the inclusion criteria. Discrepancies were resolved through discussion, with a third senior reviewer involved when necessary. Finally, the full text of 51 articles was reviewed. Thirteen RCTs were included in this meta‐analysis (Figure 1).

### 2.5 Data Extraction

After reviewing the literature, an Excel file containing the required variables was created. The extracted variables included study authors, study year, study site, study design, mean age of patients with CVD, gender distribution (male/female), smoking, diabetes, total number of study subjects, number of patients with CVD by drug group (empagliflozin and placebo), body mass index, mean initial eGFR, effect size for renal outcomes (kidney disease progression, composite renal outcome, nephropathy, doubling of serum creatinine and safety) at 95% confidence interval, CVD type, empagliflozin dose, mean duration of use and mean follow‐up period. We contacted the study authors for missing data. Data extraction was performed using two separate Excel files by two independent investigators. A third investigator resolved any discrepancies between the two investigators for a variable.

### 2.6 Quality Assessment

Risk of bias for each included RCT was assessed using the Cochrane Risk of Bias 2 (RoB 2) tool [45], which covers five domains: randomization process, deviations from intended interventions, missing outcome data, measurement of outcomes and selection of reported results. Each study was rated for risk of bias as low, some concerns or high, based on the RoB 2 guidance. Discrepancies were resolved through discussion, and we provided a summary of bias assessments across all studies using a traffic‐light plot. We also assessed certainty of evidence using the GRADE approach [46].

### 2.7 Statistical Analyses

We used Stata version 17 to analyse data with random‐effects models. We reported the pooled effect size of empagliflozin versus placebo on renal outcomes in patients with CVD as a hazard ratio (HR and 95% CI). Heterogeneity between studies was assessed with the Cochran Q and *I* ^2^ tests, and we interpreted *I* ^2^ values as follows: 0%–40% may indicate no significant heterogeneity; 40%–75% may indicate moderate heterogeneity; and > 75% indicates substantial heterogeneity. Meta‐regression was not required given the lack of heterogeneity for most outcomes or the partial heterogeneity for safety (25.5%). Also, the number of included studies for some outcomes was less than ten. Egger's test was used to assess publication bias, and the results were visually displayed in a funnel plot. Given the absence of publication bias for any of the outcomes, there was no need to use the Trim and Fill analysis to address this issue. Descriptive results were reported using tables and figures. Sensitivity analyses were performed to consider the impact of individual studies on the overall estimate of empagliflozin on outcomes.

## 3 Results

**FIGURE 2:**
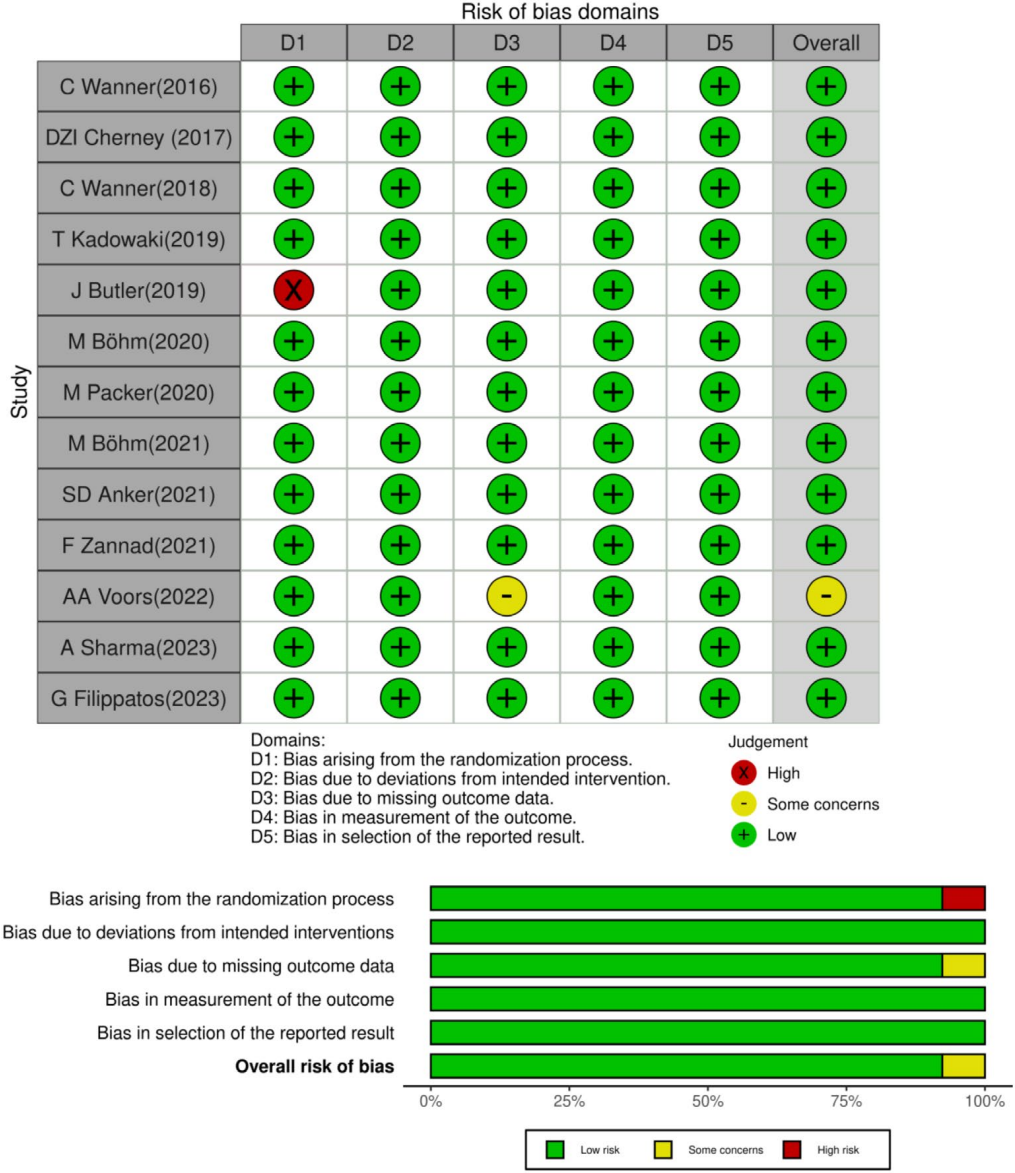
Results of risk of bias assessment by ROB2 tool.

We ultimately included thirteen RCTs [17, 25, 28, 36, 37, 38, 39, 40, 41, 47, 48, 49, 50] including 36,169 CVD patients (20,737 in the empagliflozin group and 15,432 in the placebo group). The mean age of the patients was 67.2 ± 6.6 years. Almost one‐third (32.4%) of the patients were female (32.5% and 32.4% in the Empagliflozin and placebo groups, respectively). The mean BMI was 28.7 ± 3.3 kg/m^2^ years. The mean baseline eGFR in the Empagliflozin and placebo groups was 55.3 ± 3.4 and 56.1 ± 2.8 mL/min/1.73m^2^, respectively. HF was the most common CVD. In terms of risk of bias according to the RoB 2 checklist, the majority of studies were at low risk (Figure 2). In terms of certainty of evidence, all studies were of high or moderate certainty. The median follow‐up period was 80 weeks. The baseline and demographic characteristics of the studies included in this meta‐analysis are reported in Table 1.

**TABLE 1:** Baseline characteristics, certainty of evidence and risk of bias of studies included in this meta‐analysis.

Authors (Year)	Total population	*N* of Empagliflozin/placebo	Dose	Duration (week)	Mean EGFR at baseline (Empagliflozin/placebo)	Age	Female sex (Empagliflozin/placebo)	BMI	DM (Empagliflozin/placebo)	CVD type	Certainty of evidence
Wanner [25]	1819	1212/607	10 or 25 mg	52	30.9/48.4	67.1	396/189	27.2	1212/607	CVD	High
Cherney [47]	2782	1847/935	10 mg	164	47.9/48.2	63.4	475/232	27.6	1847/935	CVD	Moderate
Wanner [28]	2250	1498/752	10 or 25 mg	96	54.5/54.3	66.1	465/223	30.8	1498/752	CVD	High
Kadowaki [48]	1517	1006/911	10 or 25 mg	164	NA	64.7	301/151	26.6	NA	CVD	Moderate
Butler [36]	706	462/244	10 or 25 mg	36	69.1/68	64.1	142/69	31.1	NA	Heart Failure	High
Böhm [49]	7020	4687/2333	10 or25 mg	164	67.1/66.4	62.8	1919/1211	30.6	NA	Atrial fibrillation	High
Packer [50]	3730	1863/1867	10 mg	52	61.8/62.2	69.9	437/456	28.5	927/929	Heart Failure	High
Böhm [38]	3726	1863/1859	10 mg	12	61.5/60.5	65.1	409/411	27.8	947/932	Heart Failure	Moderate
Anker [37]	3730	1863/1863	10 mg	52	61.3/62.7	66.9	437/458	28.02	921/929	Heart Failure	High
Zannad [39]	1978	981/997	10 mg	NA	46.5/47.4	70.2	232/273	28.01	820/981	Heart Failure	High
Voors [40]	580	265/265	10 mg	12	NA	69.4	85/84	29.3	115/112	Heart Failure	High
Sharma [17]	3198	1615/1583	10 or 25 mg	52	45.1/46.4	74.2	775/620	28.5	851/822	Heart Failure	
Filippatos [41]	3133	1575/1558	10 mg	102	63.1/62.1	69.1	655/611	29.7	758/744	Atrial fibrillation	

### 3.1 Renal Outcomes

#### 3.1.1 Progression of Kidney Disease

**FIGURE 3:**
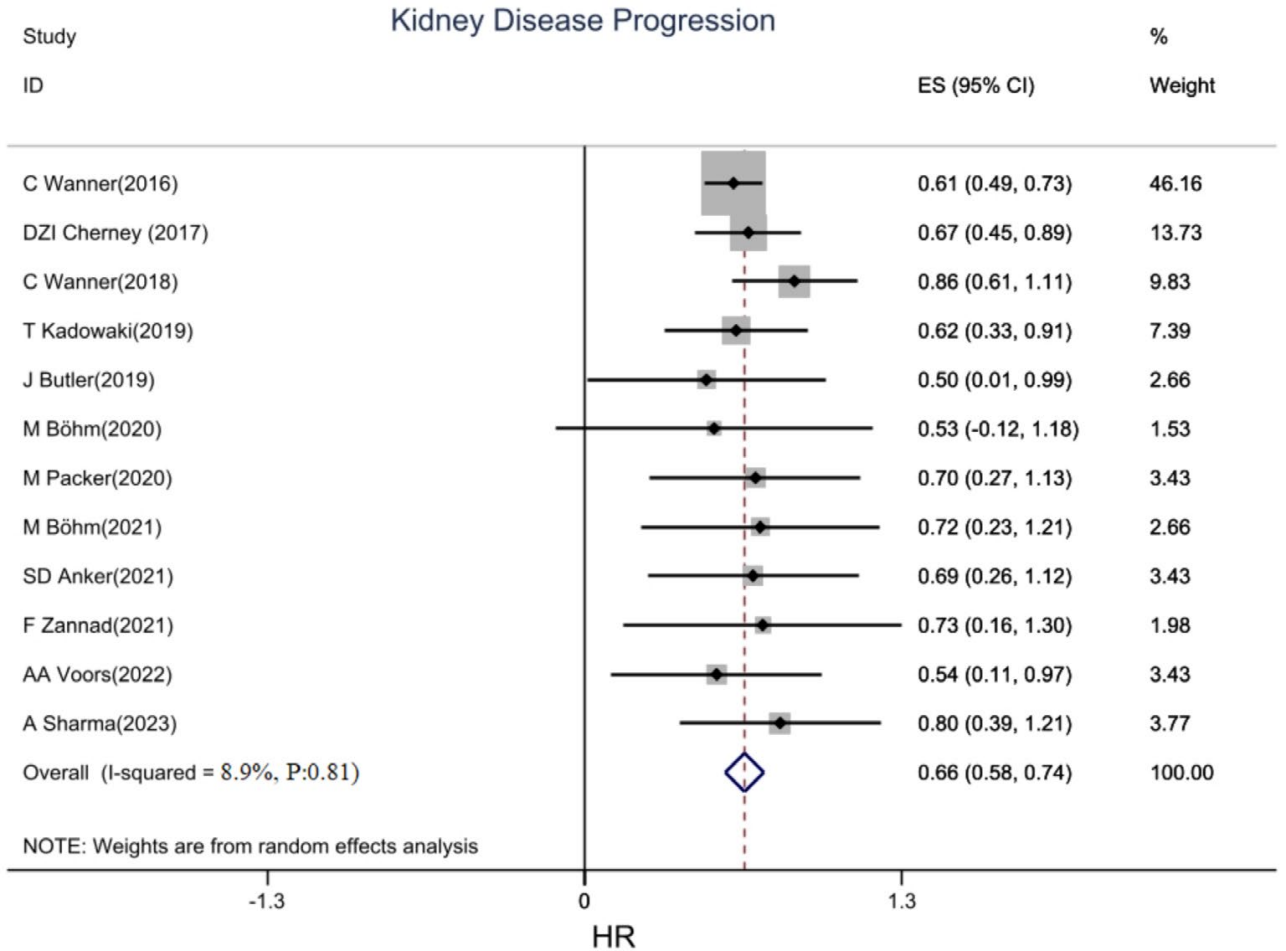
Forest plot of effect of Empagliflozin on Kidney Disease Progression in CVD patients.

A pooled analysis of 12 studies showed that empagliflozin was significantly associated with a reduced risk of kidney disease progression (HR: 0.66, 95% CI: 0.58, 0.74, I2: 11.1%) (Figure 3). Empagliflozin reduced the risk of kidney disease progression in patients with CVD and HF by 34% and 33%, respectively (Figure S1).

#### 3.1.2 Composite Kidney Outcome

**FIGURE 4:**
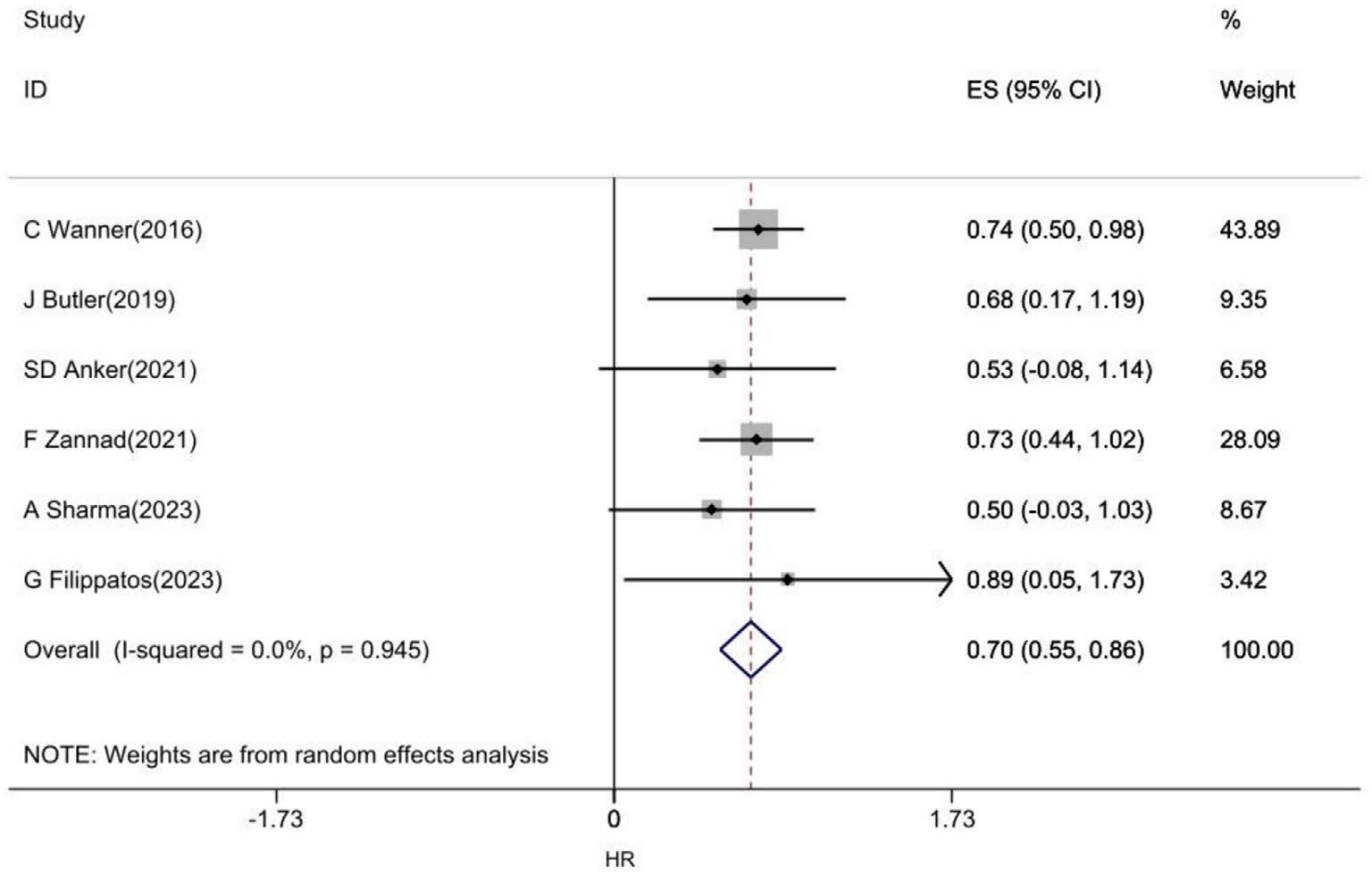
Forest plot of effect of Empagliflozin on Composite kidney outcome in CVD patients.

The association of empagliflozin with composite kidney outcome was examined in six studies [25, 36, 37, 39, 41, 50]. The pooled estimate of results showed that empagliflozin reduced the risk of composite kidney outcome by 30% (Figure 4).

#### 3.1.3 Diabetic Nephropathy

**FIGURE 5:**
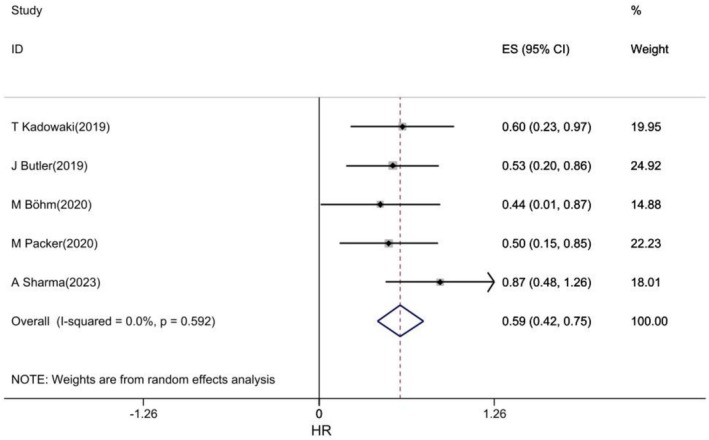
Forest plot of effect of Empagliflozin on Diabetic Nephropathy in CVD patients.

The pooled analysis of the five studies [17, 36, 48, 49, 50] showed that empagliflozin was significantly associated with a reduced risk of diabetic nephropathy in patients with CVD (HR: 0.59, 95% CI: 0.42, 0.75, I2: 0%) (Figure 5).

#### 3.1.4 Doubling of the Serum Creatinine Level

**FIGURE 6:**
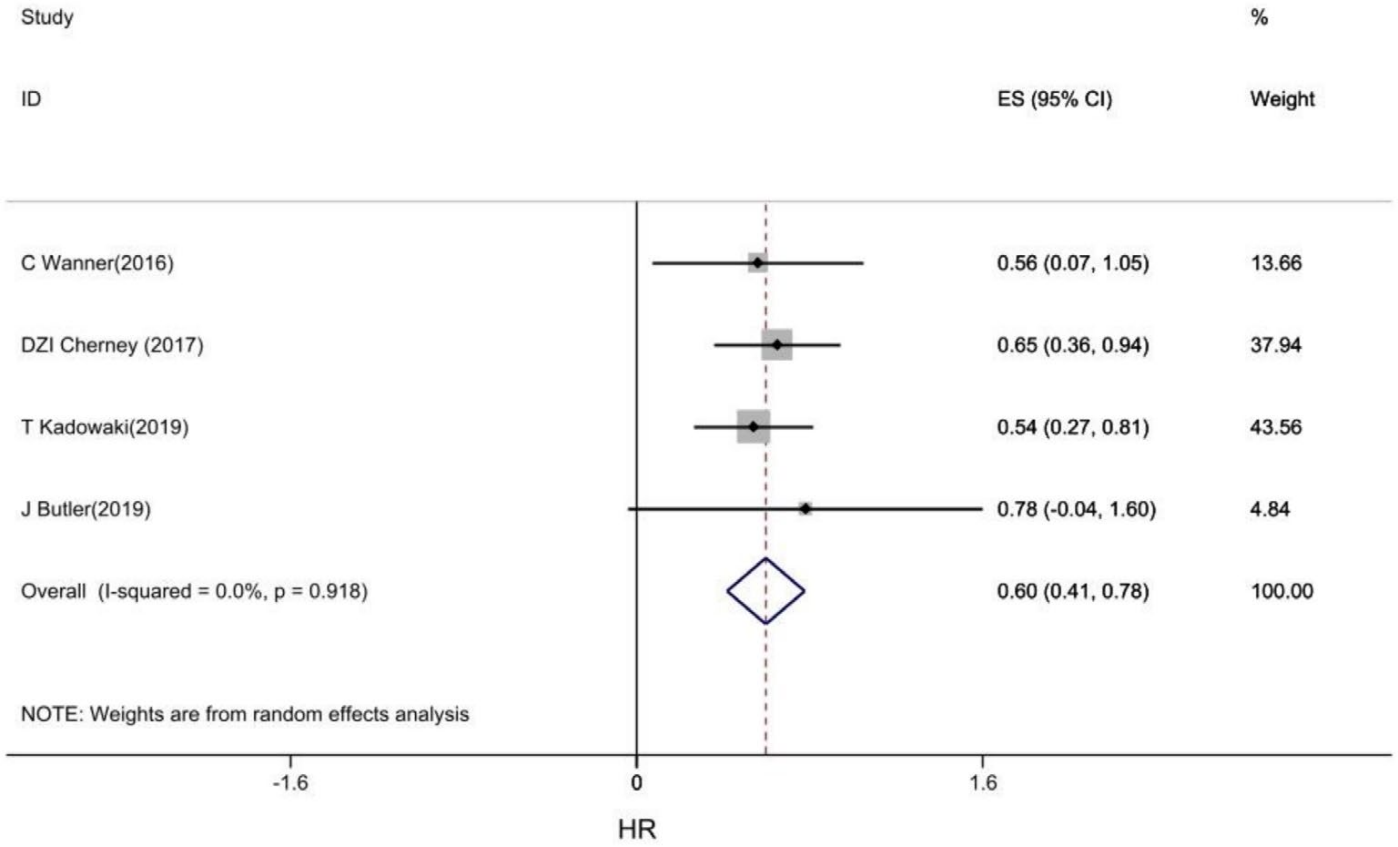
Forest plot of effect of Empagliflozin on Doubling of the serum creatinine level in CVD patients.

Four RCTs examined the effect of empagliflozin on doubling of the serum creatinine level. The pooled results showed that empagliflozin was significantly associated with a reduced risk of doubling of the serum creatinine in CVD patients, reducing the risk of doubling of the serum creatinine by 40% compared with placebo (HR: 0.60, 95% CI: 0.41, 0.78, I2: 0%) (Figure 6).

The effect of empagliflozin on replacement therapy was investigated in two studies [25, 48]. Wanner et al. [25] showed that empagliflozin reduced the risk of switching therapy by 45%. This rate was estimated to be 48% in the study by Kadowaki et al. [48].

### 3.2 Safety

**FIGURE 7:**
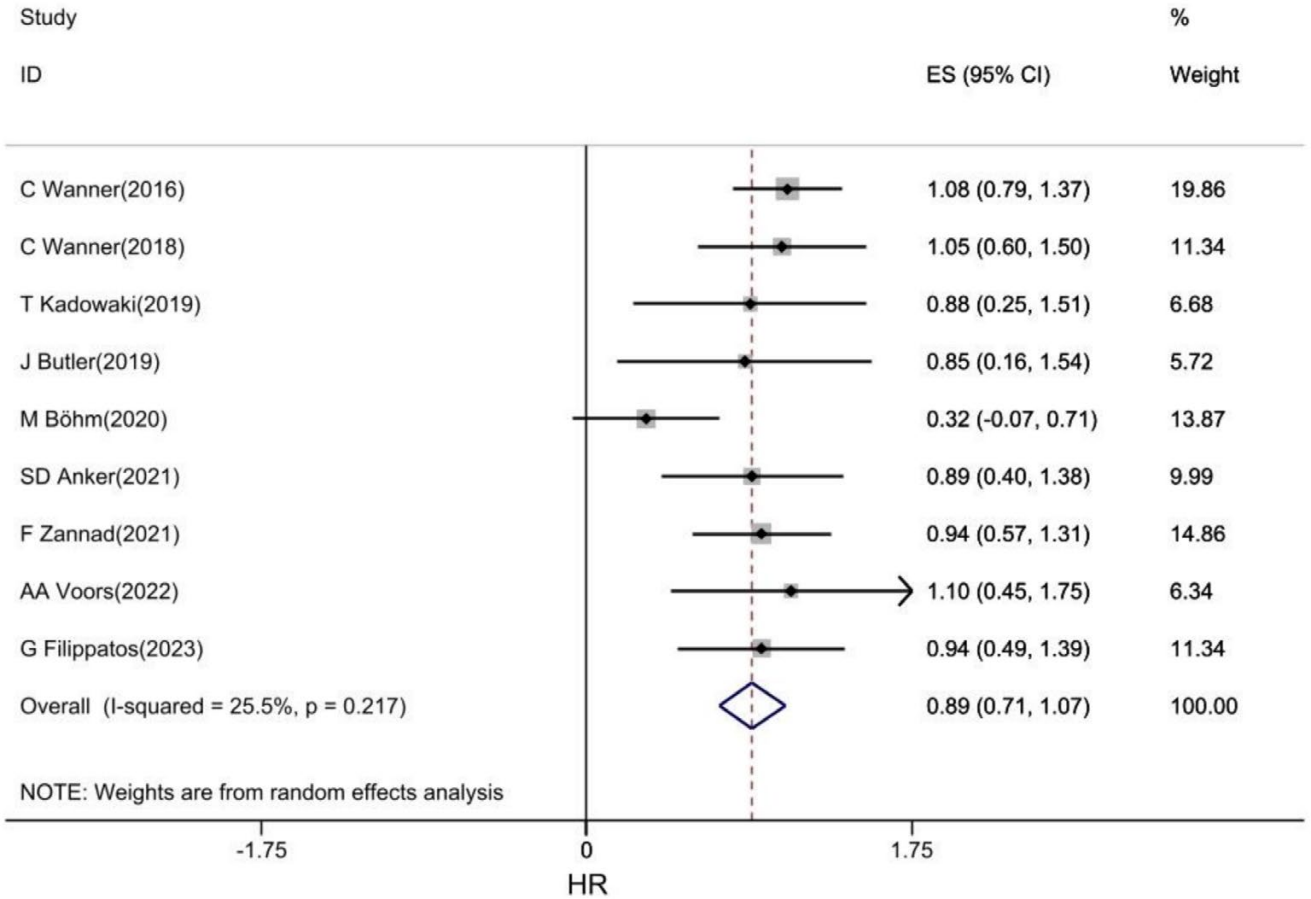
Forest plot of effect of Empagliflozin on Safety in CVD patients.

The safety of empagliflozin in patients with CVD was investigated in nine studies [25, 28, 36, 37, 39, 40, 41, 48, 49]. The pooled estimates of the results showed that empagliflozin was not significantly associated with an increased risk of any complications, especially serious complications, in patients with CVD (HR: 0.89, 95% CI: 0.71, 1.0778, I2: 25.5%) (Figure 7).

The results of subgroup analysis showed that empagliflozin was not associated with an increased risk of complications in any of the subtypes of CVD and was safe (Figure S2).

### 3.3 Sensitivity Analysis

A sensitivity analysis was performed to estimate the effect size of each study on the overall estimate of the association of Empagliflozin with kidney disease progression, composite kidney outcome and safety (Figures S3–S5).

Further analyses limited to studies that adjusted for mean eGFR, consistent with the overall estimate, also showed that empagliflozin was associated with a reduced risk of disease progression (HR: 0.55, 95% CI: 0.45, 0.66), Composite kidney outcome (HR: 0.45, 95% CI: 0.34, 0.55), Doubling of the serum creatinine level (HR: 0.52, 95% CI: 0.4, 0.63) and Diabetic Nephropathy (HR: 0.52, 95% CI: 0.40, 0.65).

### 3.4 Publication Bias

**FIGURE 8:**
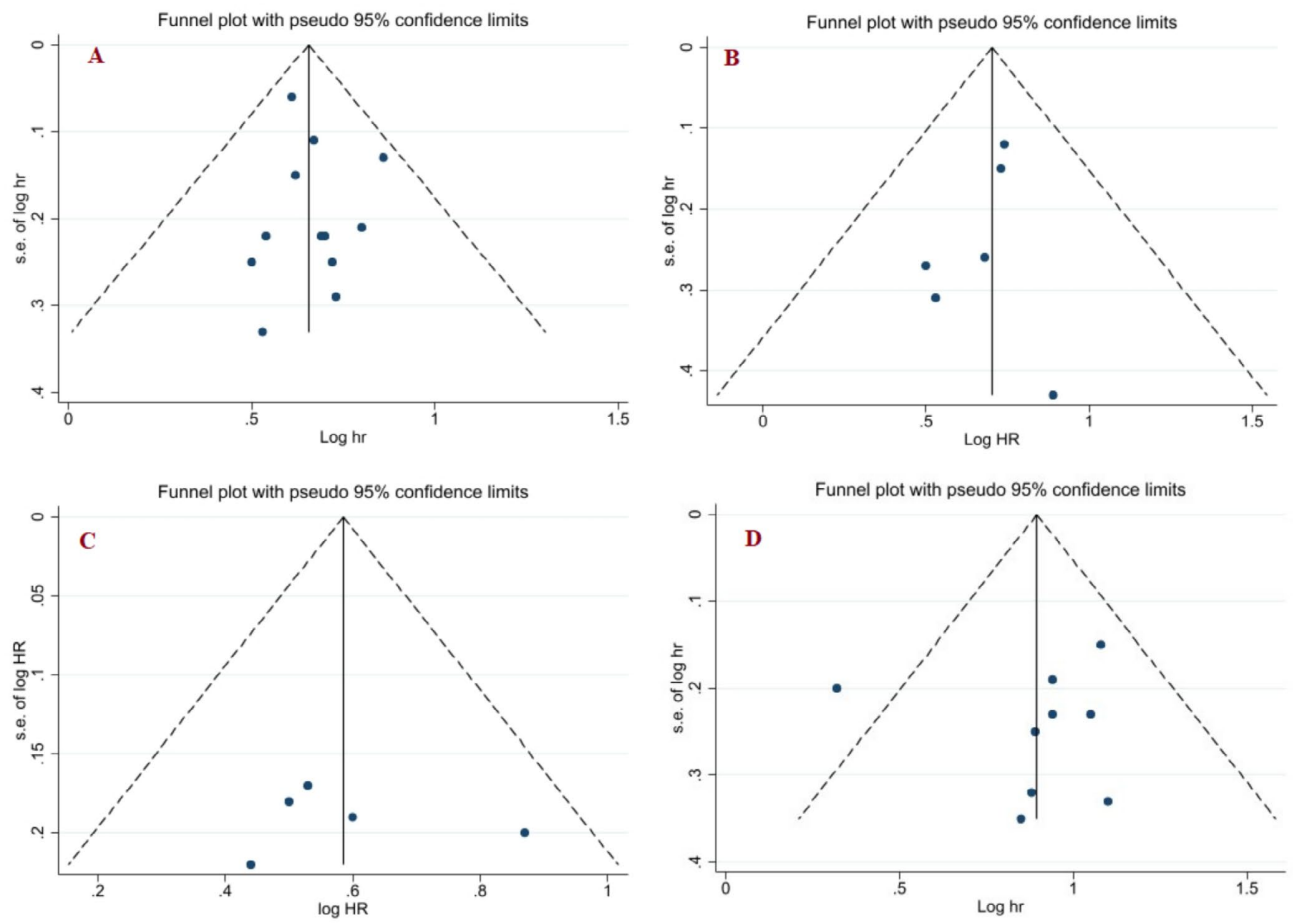
Bias publication assessment in the funnel plot for progression of kidney disease (A) composite kidney outcome (B) Diabetic nephropathy (C) and Safety (D).

We assessed the effect of publication bias by outcome using Egger's test. The results of the Egger's test showed no significant effect of publication bias on the overall estimate of the association of empagliflozin with progression of kidney disease (Egger's test: 0.24, 95% CI: −0.59, 1.08, *p*: 0.91), composite kidney outcome (Egger's test: −1.2, 95% CI: −3.2, 1.9, *p*: 0.8), Diabetic nephropathy (Egger's test: −0.51, 95% CI: 0.1.52, 0.58, *p*: 0.65) and safety in CVD patients (Egger's test: −0.23, 95% CI: −4.9, 3.5, *p*: 0.88). The distribution of studies by outcome is also reported in a funnel plot (Figure 8).

## 4 Discussion

The effect of empagliflozin on cardiac outcomes in CVD patients remains controversial. Given the importance of the topic, we pooled the results of 13 RCTs to evaluate the efficacy and safety of empagliflozin on renal outcomes in 36,169 patients with CVD with a median age of 67 years, most of whom were men (more than two‐thirds). Heart failure was the most common type of CVD.

The pooled estimate of the results showed that empagliflozin reduced the risk of kidney disease progression by 34% compared with placebo in patients with CVD, regardless of the dose (10 or 20 mg), and these results were similar across subgroups of CVD types. Empagliflozin also reduced the risk of the composite renal outcome, diabetic nephropathy and doubling of serum creatinine by 30%, 41% and 40%, respectively, in CVD patients. The effect of empagliflozin on replacement therapy was evaluated in only two studies, both of which suggested a protective effect of empagliflozin on the risk of therapy substitution (> 50%) in CVD patients. The majority of patients studied in the initial studies also had CKD or established renal impairment and empagliflozin was also significantly associated with improvement in renal indices and prevention of disease progression in CKD patients.

Pooled estimates from nine studies showed that empagliflozin was effective in CVD patients with and without established baseline CKD and did not cause any serious adverse events, including edema, renal failure, volume depletion and hyperkalemia and edema.

The risk of bias of the majority of studies included in this meta‐analysis was low based on the ROB2 tool, and the certainty of the evidence was high in the majority of studies. The heterogeneity between studies was low for all outcomes and almost zero for a number of outcomes. Given the low heterogeneity between studies, meta‐regression was not required. The small amount of heterogeneity remaining between studies for several outcomes can be explained by differences in patient demographic characteristics, comorbidities, mean follow‐up and lifestyle (nutrition and physical activity) between studies. No publication bias was observed for any of the outcomes and publication bias had no significant effect on the study results.

Although several reviews have examined the effect of SGLT2 on renal and cardiovascular outcomes in different populations [33, 51, 52, 53, 54, 55, 56, 57, 58, 59], to our knowledge, no review has yet assessed the effect of empagliflozin on renal outcomes in CVD patients. Bae et al. [60] in a systematic review and meta‐analysis evaluating the effects of SGLT 2 inhibitors on renal outcomes in patients with type 2 diabetes in 48 studies including 58,165 patients, showed that SGLT2 inhibitors were significantly associated with a reduced risk of albuminuria or its progression and a reduced risk of end‐stage renal disease compared to placebo or other diabetic drugs, which confirmed the results of our study. In another systematic review and meta‐analysis, HM Salah et al. examined the effect of SGLT2 inhibitors compared with placebo on cardiac and renal outcomes in eight trials including 59,747 patients. They showed that SGLT2i reduced the risk of the composite kidney outcome and progressive kidney disease by 38% compared with placebo in the overall population, which confirmed the results of our study. They studied the general population, while we studied only CVD patients. In another systematic review and meta‐analysis by Spiazzi et al. [58] in 2025, evaluating the effect of SGLT2i on renal outcomes across a range of kidney diseases in 10 RCTs, including 78,184 participants, SGLT2i significantly reduced the risk of renal outcomes across a range of KDIGO classes and UACR levels compared to placebo, which was in line with the results of our study. Lo et al. [55] in a systematic review and meta‐analysis evaluating the effect of SGLT2 inhibitors compared to placebo on cardiac and renal outcomes in diabetic patients, showed that SGLT2i was significantly associated with a reduced risk of renal outcomes and renal adverse events in diabetic patients compared to placebo, and these effects were evident even in a population with eGFR < 60 mL/min/1.73 m^2^.

In a systematic review and meta‐analysis, Alizadehasl et al. [54] evaluated the efficacy and safety of empagliflozin on outcomes in patients with myocardial infarction (MI) undergoing primary PCI. In a systematic review including seven RCTs, they showed that empagliflozin reduced the risk of hospitalisation for heart failure by nearly 50% compared with placebo. They also showed that empagliflozin was associated with good safety in patients with myocardial infarction.

The mechanisms of empagliflozin renal effects are likely to be multifactorial, although direct effects on the renal vasculature may play a major role [61, 62, 63]. Empagliflozin, by reducing proximal tubular sodium reabsorption, increases distal sodium delivery to the macula densa and activates tubuloglomerular feedback, thereby modulating afferent vasculature, reducing hyperfiltration and improving the progression of renal disease in patients with CVD [64]. Empagliflozin may also lead to a reduction in albuminuria and consequently an improvement in associated renal outcomes through various mechanisms, including a reduction in glomerular hyperfiltration, amelioration of tubulointerstitial fibrosis, a decrease in systemic blood pressure, changes in plasma volume expansion and a decrease in uric acid levels [61, 65, 66, 67, 68, 69, 70, 71]. In addition to its intrarenal effects, empagliflozin may affect eGFR by reducing blood pressure and body weight, thereby improving renal outcomes [72, 73]. In addition, empagliflozin can also prevent the progression of kidney disease by reducing hyperglycemia and haemoglobin A1C levels through reducing arterial stiffness and reducing vascular resistance [24, 62, 63].

### 4.1 Limitation

Our study had limitations and strengths that should be noted. Due to the lack of reporting and availability of several key variables, including duration of heart disease, smoking, comorbidities including diabetes and non‐diabetes and obesity, in the primary studies included in this meta‐analysis, we were unable to estimate the effect size of empagliflozin on renal outcomes stratified for these variables. Most of the studies included in this meta‐analysis were conducted in developed countries that had conducted randomised clinical trials with short follow‐up and caution should be exercised in generalising the results to other populations. Although the heterogeneity statistics were low, differences in study design, outcome definitions and follow‐up duration may still affect the pooled estimates. Most of the included RCTs had relatively short follow‐up periods, which limits the assessment of long‐term renal protection and safety. Several studies used a common database for different purposes, although we tried to account for data overlap, so the design of randomised clinical trials in other populations could help to estimate the results more accurately. The main objective of this study was to evaluate the efficacy and safety of empagliflozin on renal outcomes in CVD patients in a systematic review and meta‐analysis with low heterogeneity.

## 5 Conclusion

This meta‐analysis showed that empagliflozin may significantly reduce the risk of CKD progression, composite kidney outcome, diabetic nephropathy and doubling of the serum creatinine in patients with CVD compared with placebo. Empagliflozin also had a good safety profile. Empagliflozin can be prescribed as an effective and safe antidiabetic drug in patients with CVD to improve renal outcomes and prevent CKD progression. The studies included in this meta‐analysis were RCTs conducted in specific populations and specific conditions. Conducting studies in the post‐marketing phase and at the community level could help to estimate the results more accurately.

## Author Contributions

All authors contributed to the study conception and design. M.B., A.G.: Conceptualization, methodology, software, writing – original draft, data curation, visualization were performed; A.G., M.M., M.S. and B.G.: Investigation, Writing – original draft, writing – reviewing and editing were performed; A.G.: Conceptualization, supervision, project administration were performed. All authors read and approved the final manuscript.

## Funding

The authors have nothing to report.

## Ethics Statement

Protocol registered in PROSPERO.

## Conflicts of Interest

The authors declare no conflicts of interest.

## Supporting information


**Figure S1:** Forest plot of effect of Empagliflozin on Kidney Disease Progression based on CVD type.
**Figure S2:** Forest plot of effect of Empagliflozin on Safety base on CVD type.
**Figure S3:** Results of sensitivity analysis for single effect of studies on the association of empagliflozin on kidney disease progression.
**Figure S4:** Results of sensitivity analysis for single effect of studies on the association of empagliflozin on composite kidney outcome.
**Figure S5:** Results of sensitivity analysis for single effect of studies on the association of empagliflozin on safety.

## Data Availability

The data that support the findings of this study are available on request from the corresponding author. The data are not publicly available due to privacy or ethical restrictions.
