# Supplementary material for: The Effect of Empagliflozin on Renal Outcomes in Patients With Established Cardiovascular Disease: Systematic Review and Meta‐Analysis of Randomised Placebo‐Controlled Trials
Source: Endocrinol Diabetes Metab. 2026 Apr 17;9(3):e70203. doi: 10.1002/edm2.70203 (PMC13090579; doi:10.1002/edm2.70203)

Supplementary Figure1: Forest plot of effect of Empagliflozin on Kidney Disease Progression based on CVD type.


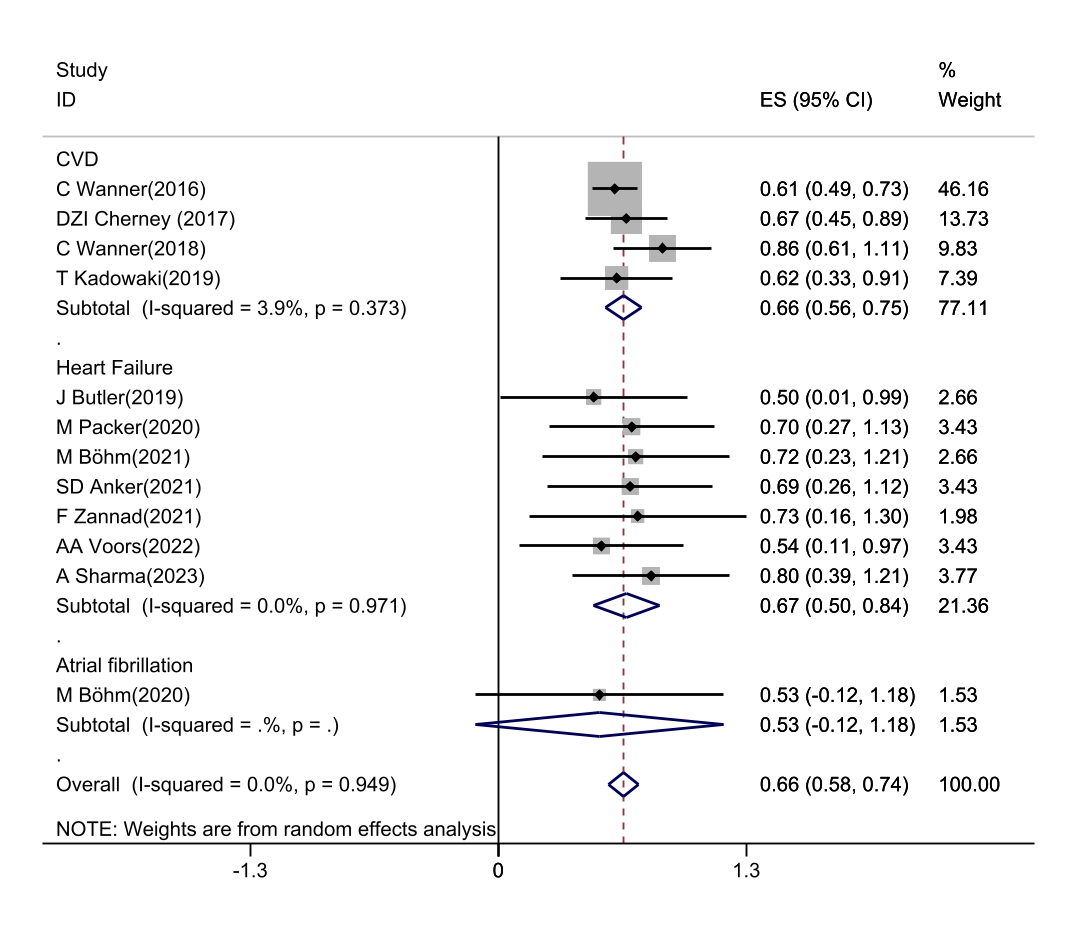


Supplementary Figure 2: Forest plot of effect of Empagliflozin on Safety base on CVD type.


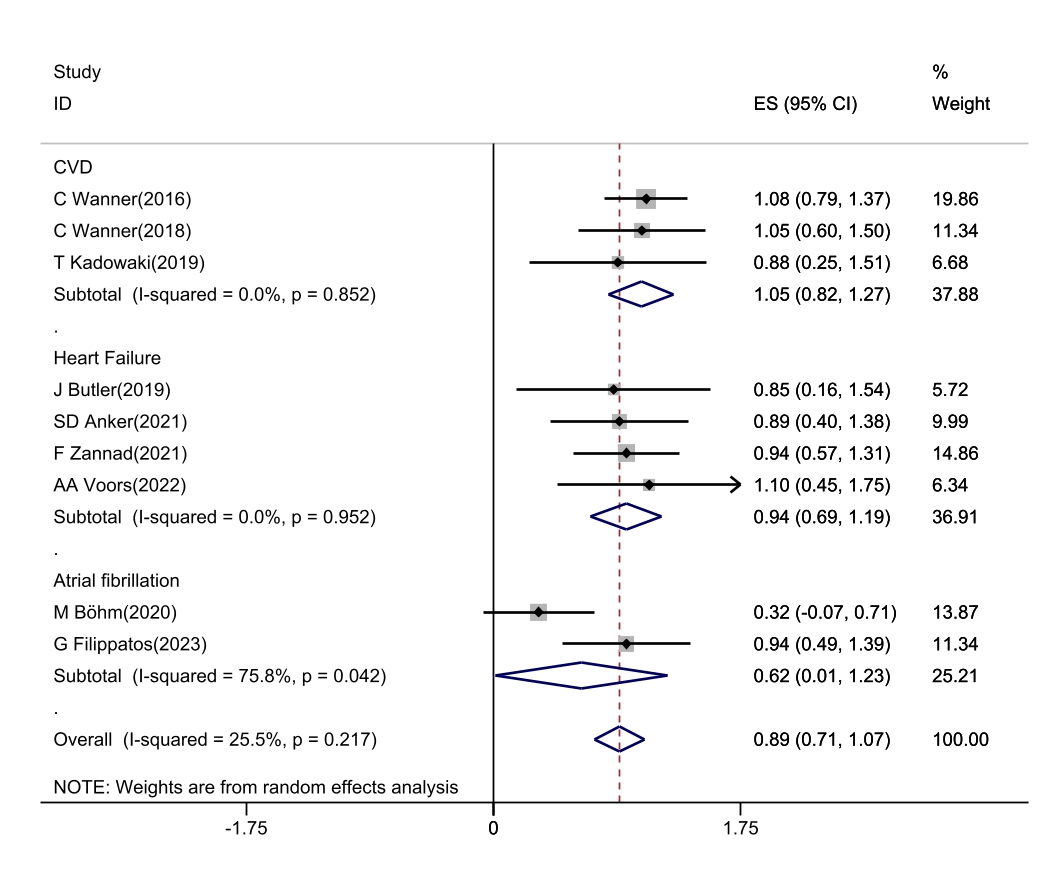


Supplementary Figure 3: Results of sensitivity analysis for single effect of studies on the association of empagliflozin on kidney disease progression


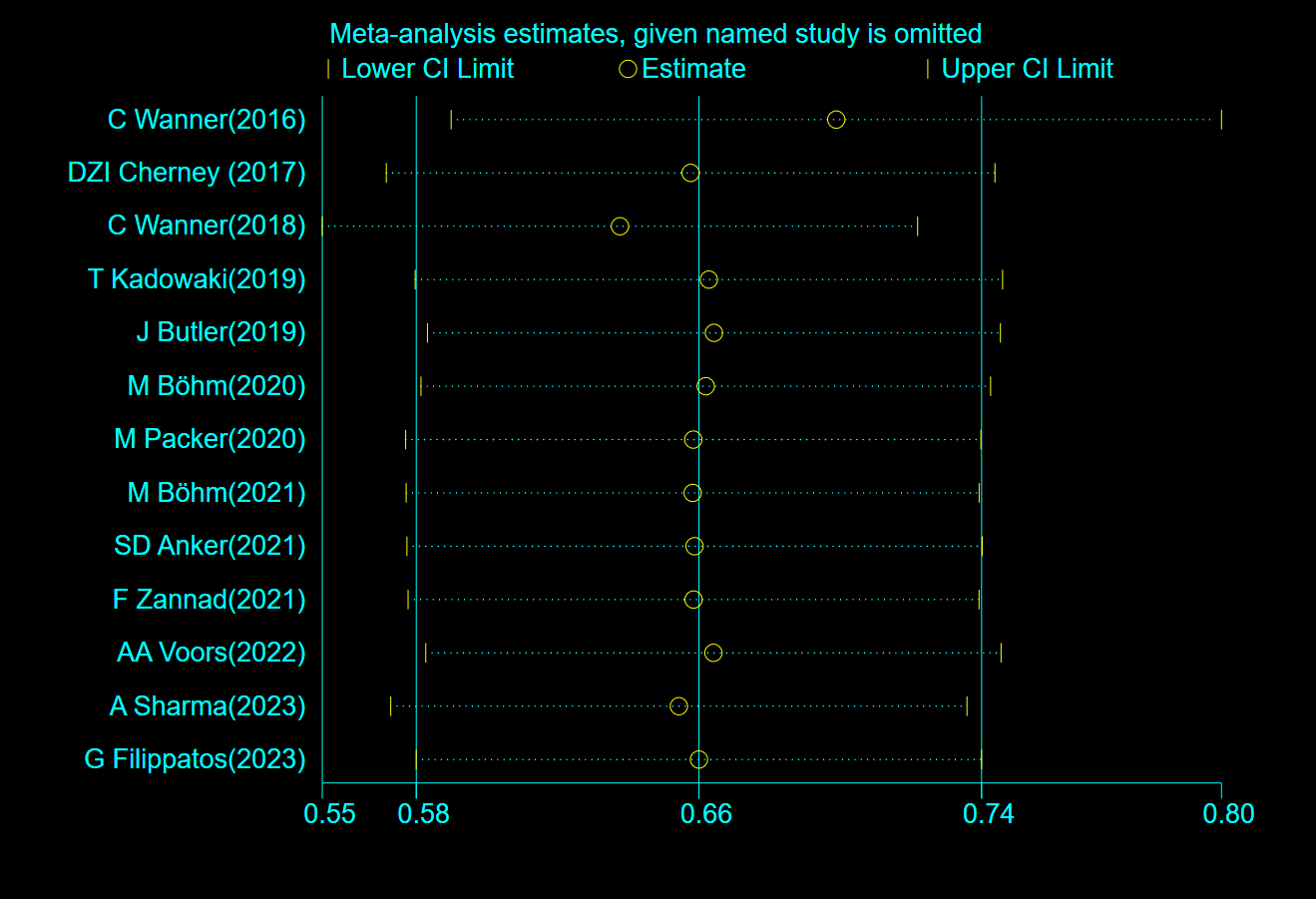


Supplementary Figure 4: Results of sensitivity analysis for single effect of studies on the association of empagliflozin on composite kidney outcome


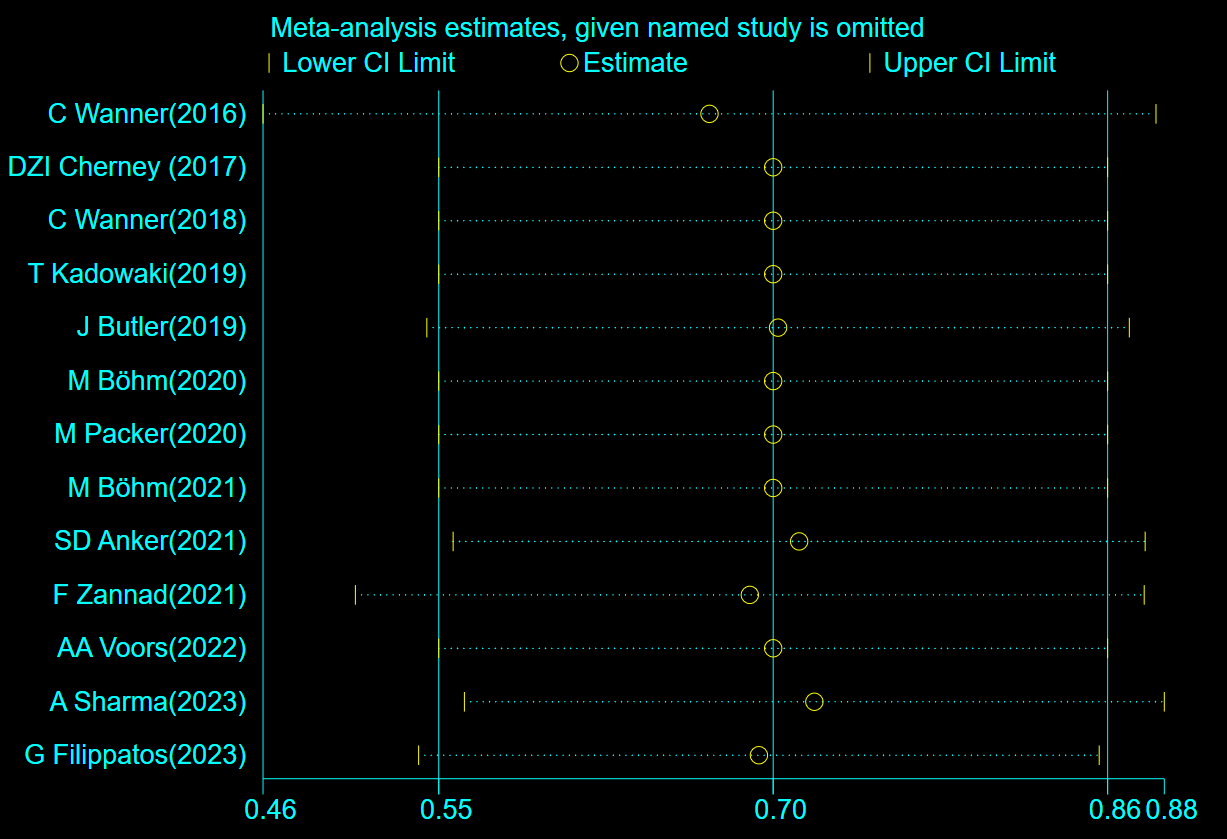


Supplementary Figure 5: Results of sensitivity analysis for single effect of studies on the association of empagliflozin on safety


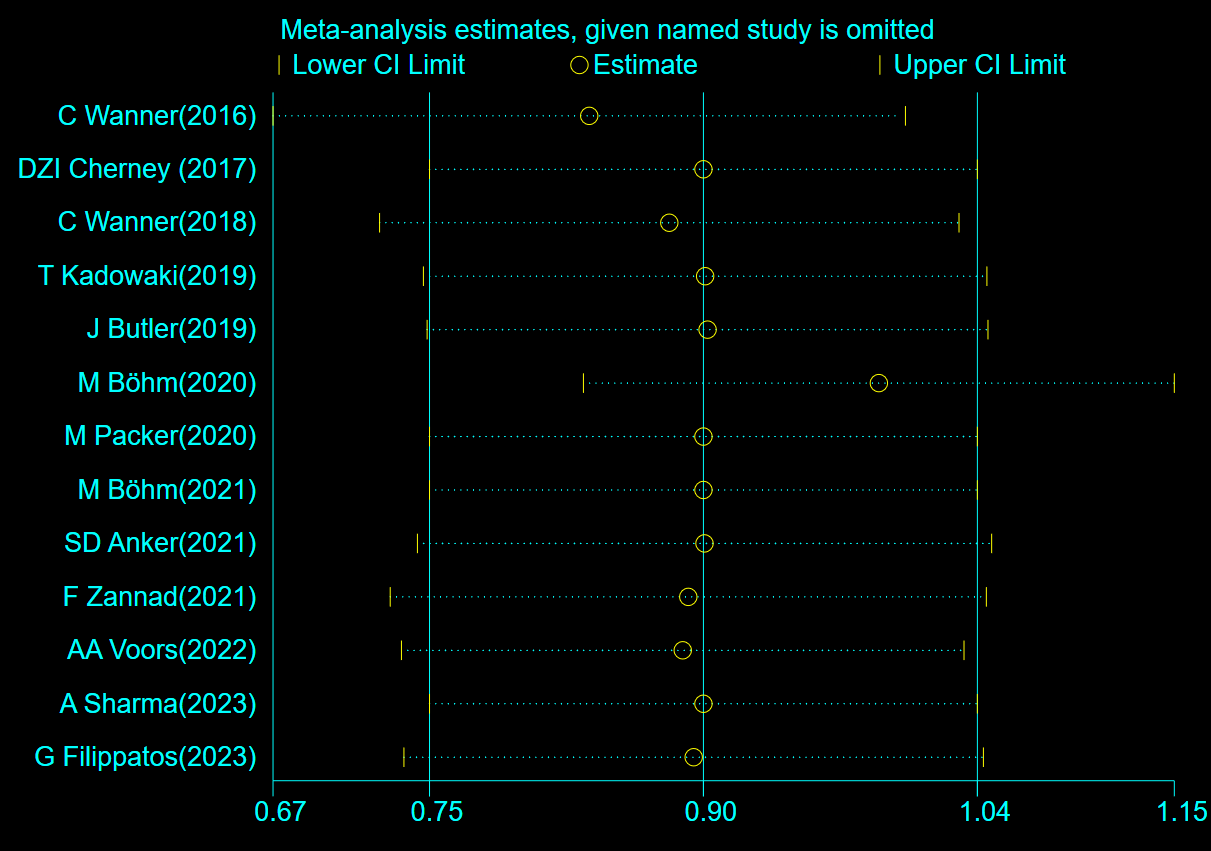

Supplement: Supplementary file 1 — Figure S1: Forest plot of effect of Empagliflozin on Kidney Disease Progression based on CVD type. Figure S2: Forest plot of effect of Empagliflozin on Safety base on CVD type. Figure S3: Results of sensitivity analysis for single effect of studies on the association of empagliflozin on kidney disease progression. Figure S4: Results of sensitivity analysis for single effect of studies on the association of empagliflozin on composite kidney outcome. Figure S5: Results of sensitivity analysis for single effect of studies on the association of empagliflozin on safety. [file EDM2-9-e70203-s001.docx]
